# Imaging characterization of myocardial function, fibrosis, and perfusion in a nonhuman primate model with heart failure-like features

**DOI:** 10.3389/fcvm.2023.1214249

**Published:** 2023-08-17

**Authors:** Xing-Li Liu, Guan-Zhong Wang, Mao-Ping Rui, Dong Fan, Jie Zhang, Zheng-Hua Zhu, Rosario Perez, Tony Wang, Li-Chuan Yang, Liang Lyu, Jie Zheng, Gang Wang

**Affiliations:** ^1^ Department of Radiology, The First People’s Hospital of Yunnan Province, Kunming, China; ^2^ Department of Radiology, The Affiliated Hospital of Kunming University of Science and Technology, Kunming, China; ^3^ Department of Pharmocolgy, Kunming Biomed International of TriApex Group, Kunming, China; ^4^ Mallinckrodt Institute of Radiology, Washington University in Saint Louis, St. Louis, MO, United States

**Keywords:** heart failure, nonhuman primate, cardiovascular magnetic resonance, perfusion, extracellular volume, fibrosis

## Abstract

**Introduction:**

The availability of a human-like chronic heart failure (HF) animal model was critical for affiliating development of novel therapeutic drug treatments. With the close physiology relatedness to humans, the non-human primate (NHP) HF model would be valuable to better understand the pathophysiology and pharmacology of HF. The purpose of this work was to present preliminary cardiac image findings using echocardiography and cardiovascular magnetic resonance (CMR) in a HF-like cynomolgus macaque model.

**Methods:**

The NHP diet-induced model developed cardiac phenotypes that exhibited diastolic dysfunction with reduced left ventricular ejection fraction (LVEF) or preserved LVEF. Twenty cynomolgus monkeys with cardiac dysfunction were selected by echocardiography and subsequently separated into two groups, LVEF < 65% (termed as HFrEF, *n* = 10) and LVEF ≥ 65% with diastolic dysfunction (termed as HFpEF, *n* = 10). Another group of ten healthy monkeys was used as the healthy control. All monkeys underwent a CMR study to measure global longitudinal strain (GLS), myocardial extracellular volume (ECV), and late gadolinium enhancement (LGE). In healthy controls and HFpEF group, quantitative perfusion imaging scans at rest and under dobutamine stress were performed and myocardial perfusion reserve (MPR) was subsequently obtained.

**Results:**

No LGE was observed in any monkey. Monkeys with HF-like features were significantly older, compared to the healthy control group. There were significant differences among the three groups in ECV (20.79 ± 3.65% in healthy controls; 27.06 ± 3.37% in HFpEF group, and 31.11 ± 4.50% in HFrEFgroup, *p* < 0.001), as well as for stress perfusion (2.40 ± 0.34 ml/min/g in healthy controls vs. 1.28 ± 0.24 ml/min/g in HFpEF group, *p* < 0.01) and corresponding MPR (1.83 ± 0.3 vs. 1.35 ± 0.29, *p* < 0.01). After adjusting for age, ECV (*p* = 0.01) and MPR (*p* = 0.048) still showed significant differences among the three groups.

**Conclusion:**

Our preliminary imaging findings demonstrated cardiac dysfunction, elevated ECV, and/or reduced MPR in this HF-like NHP model. This pilot study laid the foundation for further mechanistic research and the development of a drug testing platform for distinct HF pathophysiology.

## Introduction

Heart failure (HF), with either reduced (HFrEF) or preserved ejection fraction (HFpEF), is an increasingly prevalent condition affecting over 64 million people worldwide (1). Typical symptoms and signs may not be present in the early stages of HF, especially in HFpEF. Few interventions have been shown to significantly impact survival in patients with HF (2, 3). Even the most effective treatments only slow the progression of the disease without providing long-term survival benefits and have significant adverse side effects (4). Thus, it is an irrefutable need to investigate the pathophysiology of the disease and to develop more effective medical treatments (5).

Animal models have played a significant role in the development of putative treatments. For translational applications, there was a strong need for continuous improvement of clinically relevant large animal models, especially in human-like primates. Although several other large animal models of HF have been reported recently, few of them have been able to achieve satisfaction in replicating the pathophysiological state of human HF (6). A lack of preclinical models of HF in non-human primates (NHP) was one of the main barriers to developing novel effective treatments for HF (7). In comparison to rodents, the NHP model mimicked human pathophysiology and could be outfitted with more comprehensive imaging and sampling protocols, providing a better understanding of the pathophysiology and pharmacology in drug development. To date, several NHP models of metabolic dysfunction have been reported, with all of them focusing on diastolic function (8–10). A NHP model that mimics HF features was recently developed by Kunming Bio-med International (KBI). The purpose of this preliminary study was to characterize the cynomolgus monkey model with HF-like features that closely approximate the adult human HF phenotype. Both echocardiography and cardiovascular magnetic resonance (CMR) were used to non-invasively assess these features.

## Methods

### Animal model

The HF-like model was induced by a high-fat diet. Male monkeys were divided into the HF group and the Healthy group. Both groups were supplied with 100 g of a normal diet for breakfast, and 150 g of fruit for lunch every day. A high-fat diet feeding was arranged at dinner, where the HF group was fed 100 g of a high-fat diet and the control group was provided with 100 g of a normal diet. The two diets have been formulated as follows: (1) High-fat diet: Carbohydrate 58.6 g/100 g; Energy 418 kcal/100 g (including protein, fiber, and minerals), Vitamin B12: 47.1 μg/100. Fat: 15% of the diet. (2) Normal diet: Carboydrate 58.3 g/100 g; Energy 363 kcal/100 g (including protein, fiber, and minerals), Vitamin B12: 109 μg/100. Fat: 4.8% of the diet. The main component of fat was lard, which was mainly composed of saturated fatty acids and cholesterol. During the diet-induced period, echocardiography screening was conducted and the modeling was considered to be complete when the diagnostic criteria for HFpEF or HFrEF were met. Subsequently, a CMR examination was conducted to further characterize the myocardial tissue in these monkeys.

**Figure 1:**
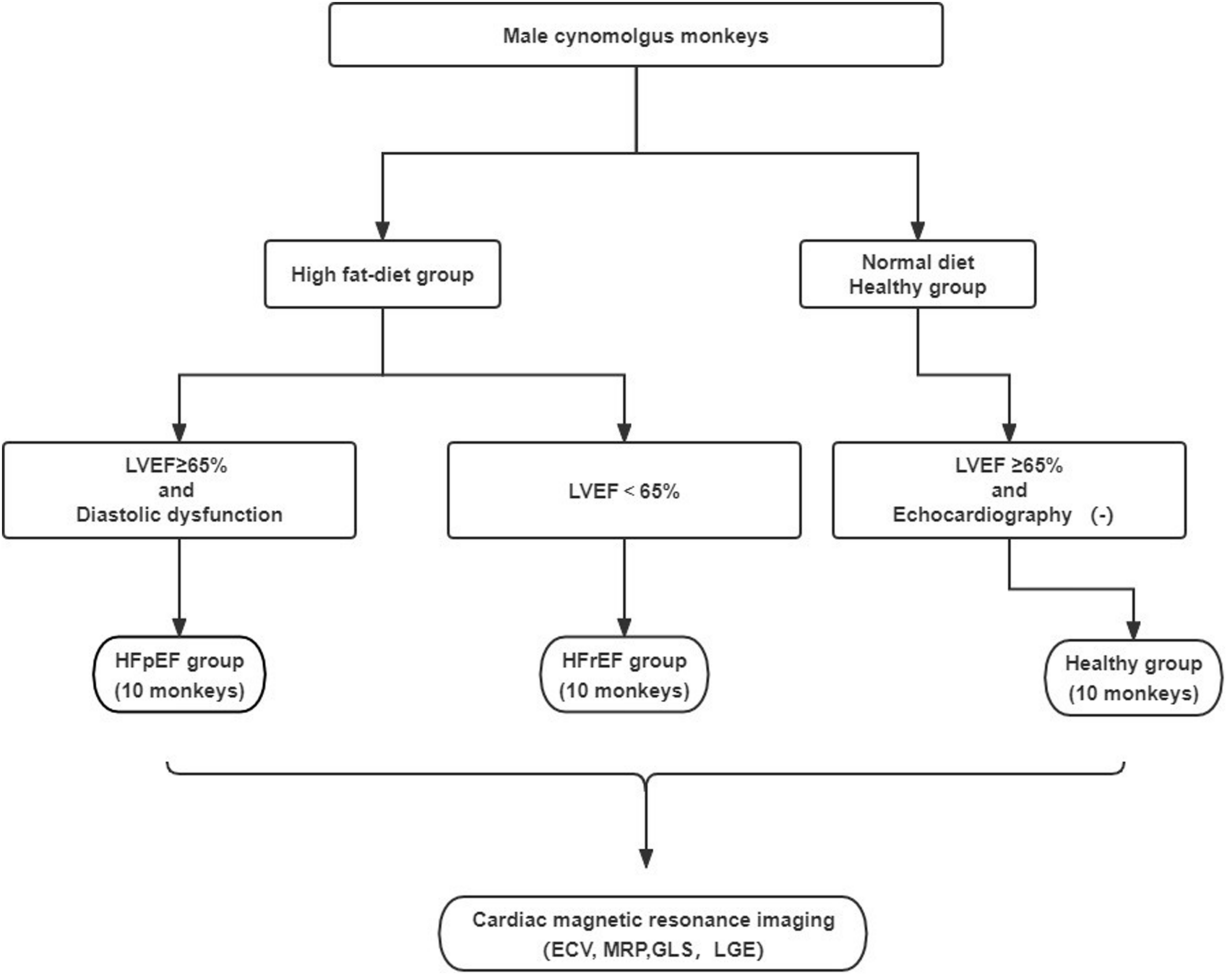
Flow chart of animal model establishment and evaluation.

Twenty monkeys with cardiac dysfunction based on echocardiogram were identified as the HF-like group. These monkeys were further classified into two groups: EF ≥ 65% with diastolic dysfunction as HF with preserved EF (termed as the HFpEF group). Diastolic dysfunction was determined if at least two echocardiography criteria were met: (1) *E*′/*A*′ or *E*/*A* ratio < 0.8 or >3; (2) septal *E*′ < 7 (cm/sec); (3) *E*/*E*′ ratio > 10. Ejection fraction (EF) < 65% as HF with reduced EF (termed as the HFrEF group). Additionally, 10 normal cynomolgus monkeys on a regular diet were included as the healthy control group. The animal model selection process was illustrated in Figure 1.

The monkeys were maintained according to guidance of National Institutes of Health Guide for the Care and Use of Laboratory Animals and the Association for Assessment and Accreditation of Laboratory Animal Care. All experimental protocols were reviewed and approved by the Experimental Animal Ethics Committee of KBI.

### Serum biochemistry measurements

A peripheral blood sample was collected for serum biochemistry and hematocrit measurements prior to an echocardiogram and CMR examination.

### Echocardiography

Transthoracic echocardiography was performed using a standard protocol with a diagnostic ultrasound system (Mindray M9CV, China) (11). All monkeys were first placed in a dorsal decubitus position and sedated with propofol at 4–10 mg/kg/h intravenously. Following the recommendations of the American Society of Echocardiography, all echocardiographic images from three or more consecutive cardiac cycles were digitally stored for offline analysis. Pulsed-wave Doppler measurements were conducted to record the velocity of blood flow at the mitral diastolic inflow, which was located at the level of the mitral leaflet tips, as seen from the apical 4-chamber view. The mitral early diastolic peak velocity of the *E* wave, the late peak velocity of the *A* wave, and the *E*/*A* ratio were recorded and registered by standard echocardiography. To measure the *EF* value, conventional M-mode Doppler ultrasound was used to acquire real-time two-dimensional targeted echocardiograms of the left ventricle's minor axis at the papillary muscle level. Tissue Doppler imaging was employed from the apical four-chamber view at the septal side of the mitral annulus. Early (*E*′) and late (*A*′) diastolic mitral annulus peak velocities and the ratio of early to late peak velocities (*E*′/*A*′) were then obtained.

### CMR acquisition

**Figure 2:**
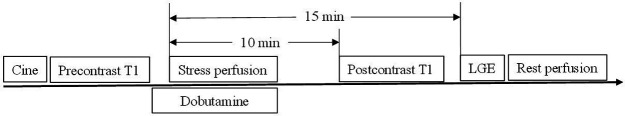
CMR imaging protocol in monkeys with dobutamine perfusion involved.

The CMR examinations were performed in a 1.5T scanner (Magnetom Aera, Siemens Healthcare) with a body phase-array and spine coils on the scanning table as the signal receivers. All monkeys underwent CMR with the following imaging protocol: (1) cine imaging along 2-, 3-, and 4-chamber views; (2) pre- and post-contrast T1 mapping; and (3) late gadolinium enhancement (LGE) on one mid-short-axis slice. Additionally, both in the HFpEF group and the healthy control group, first-pass perfusion imaging scans at rest and under dobutamine stress were performed to quantitatively evaluate myocardial perfusion and perfusion reserve. The perfusion examination was not carried out in monkeys with HFrEF for safety reasons. Figure 2 shows the CMR procedures in monkeys with perfusion imaging.

For each perfusion imaging, a double-bolus method was adopted: a 0.01 mmol/kg and 0.09 mmol/kg MRI contrast agents were administrated (Gadoteric Acid Meglumine, HENRUI Healthcare) with a flow rate of 1 ml/s and flushed with 10 ml of saline at the same flow rate. The monkey was held in breath-holding by briefly stopping airflow through the tracheal tube for the initial 15 s of the perfusion scan and then resumed normal breathing. To induce dobutamine stress, dobutamine was titrated at 5 μg/kg/min increments every 3 min until a maximum of 40 μg/kg/min was reached, or until the heart rate (HR) reached at least 25% higher than the resting HR, followed by stress perfusion imaging.

For myocardial native T1 measurements, mid ventricular short-axis images were acquired by the modified Look-Locker inversion recovery (MOLLI) 5(3)3 acquisition scheme sequence after local shimming. Post-contrast MOLLI T1-mapping was performed 15–20 min after the administration of Gd-DTPA, using a MOLLI 4(1)3(1)2 acquisition scheme sequence on the same short-axis location. This study has adjusted the parameters of the T1 mapping sequence to adapt to a relatively high heart rate in monkeys (∼150 beats per min), by increasing the number of recovery heartbeats between two groups of T1-weighted data acquisitions. The LGE scan was performed approximately 15 min after the injection of the contrast agent, followed by the rest of the perfusion imaging. All imaging parameters are shown in Table 1.

**Table 1:** CMR imaging parameters.

Sequences	Cine (TrueFISP)	Perfusion	T1 mapping	LGE
Field of view read (mm)	140	180	130	130
Field of view phase (mm)	140	135	130	130
Slice thickness (mm)	5	5	5	5
Acquired pixel size (mm^2^)	1.5 × 1.5	1.6 × 1.6	1.0 × 1.0	1.2 × 1.2
Matrix size (read × phase)	96 × 96	112 × 84	128 × 128	112 × 112
Temporal resolution (ms)	21.6	None	None	None
TR (ms)/TE (ms)	2.6/1.3	2.1/1.2	2.7/1.35	2.5/1.3
Averages	1	1	1	—
Number of TI/mode	None	None	Pre-contrast: MOLLI 5(3)3; post-contrast: MOLLI 4(1)3(1)2	1
GRAPPA factor	2	2	2	2
Partial fourier	None	None	6/8	None
Bandwidth (Hz/Px)	775	645	590	910
Scan time (s)	4 s	70 heartbeats	17 s	4 s
Flip angle	60	12	35	45

LGE, late gadolinium enhancement; MOLLI, modified Look-Locker inversion recovery; GRAPPA, GeneRalized autocalibrating partial parallel acquisition.

### Image and data analysis

Myocardial global longitudinal strains (GLS) were calculated using a commercial Medis software (Medis Medical Imaging, Leiden, the Netherlands), using 2-, 3-, and 4-chamber cine images. Myocardial extracellular volume (ECV) of the mid slice was calculated from the pre- and post-contrast T1 values with the hematocrit obtained from the serum (12). A custom-made software written in MATLAB (MathWorks, Natick, MA) was used to create ECV maps. Then the global ECV value was obtained by drawing a ROI on the entire myocardial region of the left ventricle of the ECV map of the middle short-axis slice. Myocardial perfusion was quantified using a custom-made software with the Fermi-deconvolution method, and perfusion maps on a pixel-by-pixel basis were created (13, 14). In the HFpEF group and the healthy control group, myocardial perfusion reserve (MPR) was calculated as the ratio of perfusion during the dobutamine stress to perfusion at rest. All MRI data were analyzed by two experienced radiologists (X.L., J.Z.) blinded to the echocardiography to achieve consensus for final quantitative measurements.

### Statistical analysis

The data was presented as the mean ± SD. Comparisons of CMR parameters and echocardiographic parameters among the three groups were performed using one-way ANOVA. Correlations among ECV, MPR, GLS, and LVEF were performed with a linear regression. An analysis of covariance and partial correlation was used for the age adjustment. In addition, the diagnostic efficacy of ECV for different groups of monkeys was assessed by a nonparametric receiver operating characteristic (ROC) curve. The cutoff values were determined based on the Youden index, and the areas under the curves (AUCs) were obtained. A *p*-value of less than 0.05 indicates statistical significance. Statistical analysis was performed using SPSS (Version 22, IBM) and MedCalc (Version 18, MedCalc).

## Results

### Demographic and metabolic profile

The basic demographic and metabolic profile of all monkeys was listed in Table 2. Compared to the healthy controls, the monkeys with HF were significantly older, and the two phenotypes of monkeys were all selected from many monkeys with high-fat diets, without any difference in diet, age, or diet intervention. Therefore, it appeared that two phenotypes had been developed naturally. The HbA1c percentage, LDL, and CHO level in the HF-like group were significantly higher than those in the healthy control group. (8.79 ± 4.31 vs. 5.58 ± 4.40, *p* = 0.01 for HbA1c%; 6.91 ± 4.81 vs. 3.84 ± 2.32 for CHO, *p* = 0.04; 4.54 ± 1.80 vs. 2.54 ± 1.90 for LDL *p* = 0.01). A HbA1c (%) level greater than 4.5% was considered as diabetes in monkeys (15). In our cohorts, the incidence of diabetes was 80% in the HFpEF group and 60% in the HFrEF group.

**Table 2:** Demographic and metabolic information of three groups of monkeys.

	Healthy group	HFpEF group	HFrEF group	*p*-Value
Age (years)	9.6 ± 1.3	19.9 ± 1.4^a^	16.2 ± 2.7^a^	<0.001
BW (Kg)	8.17 ± 1.98	9.02 ± 1.10	9.76 ± 2.18	0.187
High-fat diet duration (days)	0	986 ± 777	866 ± 526.25	0.248
Hct (%)	51.40 ± 6.5	51.49 ± 5.0	50.83 ± 5.0	0.947
HbA1c (%)	4.49 ± 1.07	8.2 ± 3.34^a^	9.45 ± 5.32^a^	0.045
HDL (mmol/L)	1.48 ± 0.53	1.34 ± 0.71	1.2 ± 0.66	0.232
LDL (mmol/L)	2.54 ± 1.90	3.57 ± 1.72^a,b^	5.5 ± 1.37^a,b^	0.011
CHO (mmol/L)	3.84 ± 2.32	5.43 ± 2.80	7.8 ± 2.66^a^	0.020
Triglyceride (mmol/L)	1.21 ± 1.55	5.76 ± 7.84	7.49 ± 11.53	0.152
BUN (mmol/L)	5.42 ± 1.77	5.37 ± 1.08	5.34 ± 1.74	0.259
Creatinine (μmol/L)	97.67 ± 15.99	84.14 ± 21.69	84.7 ± 17.1	0.247
Diabetes (Number)	3	8	6	0.378
Systolic pressure (mmHg)	106	120	114	0.060
Diastolic pressure (mmHg)	60	71	50	0.174
Hypertension (Number)	0	2	1	0.377

BW, body weight; Hct, red blood cell specific volume; HbA1c, hemoglobin A1C; HDL, high-density lipoprotein; LDL, low-density lipoprotein; CHO, carbohydrate; BUN, blood urea nitrogen; LVEF, left ventricular ejection fraction; EDV, end diastolic volume; ESV, end systolic volume.

^a^ Comparison with healthy controls, *p* < 0.05.

^b^ Comparison between HFpEF and HFrEF, *p* < 0.05.

### Echocardiography findings

**Figure 3:**
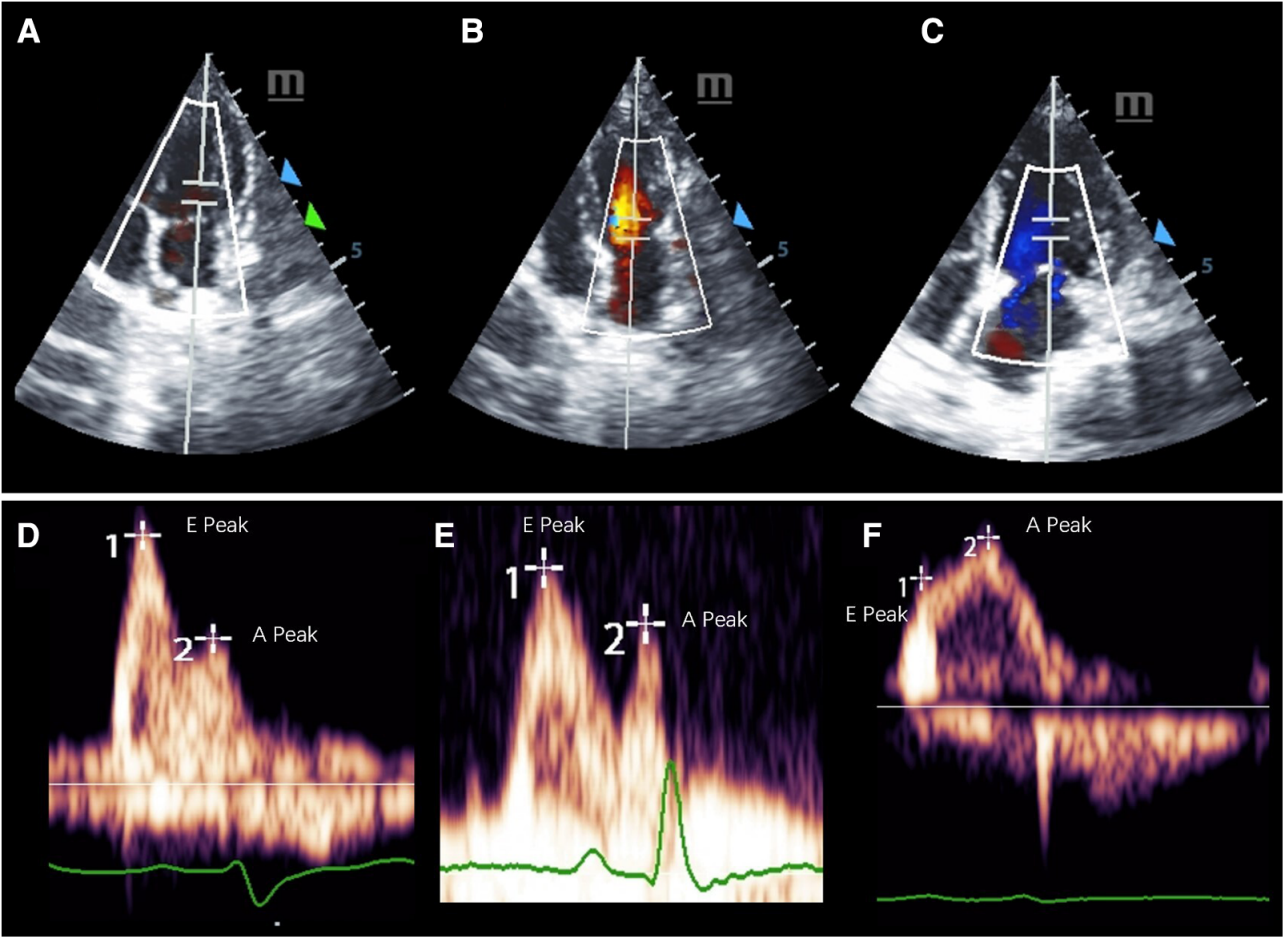
Representative pulsed-wave Doppler images of mitral inflow in three groups of cynomolgus monkeys. (**A–C**) shows the measurement location and the (**E**,**F**) display the E and A peaks of each group.

M-mode Doppler ultrasound was used to measure the left ventricular contractile capacity. There was no significant difference in LVEF between the healthy control and the HFpEF groups. However, the LVEF value of the HFrEF group decreased significantly, which was similar to that of human patients with systolic dysfunction. A decreasing *E*′/*A*′ or *E*/*A* ratio and low *E*′ velocity was the typical echocardiography finding with diastolic dysfunction (Table 3). Pulse Doppler for *E*, *A* peak measurement were shown in Figure 3.

**Table 3:** Echocardiography and CMR results.

	Measurement indicator	Healthy group	HFpEF group	HFrEF group	*p*-Value
Echocardi-ography	*E*/*A*	1.26 ± 0.23	0.69 ± 0.03^c^	0.82 ± 0.22^c^	0.001
	*E*′/*A*′	1.16 ± 0.25	0.77 ± 0.16^c^	0.67 ± 0.12^c^	0.001
	Septal *E*′	9.50 ± 2.31	4.78 ± 0.77^c^	5.02 ± 1.54^c^	<0.001
	*E*/*E*′	8.04 ± 2.2	11.49 ± 1.42^c^	10.1 ± 2.36^c^	0.004
	EDV (ml)	10.33 ± 3.38	9.35 ± 2.54	11.63 ± 3.41	0.639
	ESV (ml)	3.18 ± 1.43	2.6 ± 1.64	5.73 ± 3.4	0.232
	LVEF (%)	73.96 ± 4.55	76.64 ± 8.72	47.99 ± 8.95	<0.001
CMR	GLS (%)	−22.66 ± 2.15	−20.70 ± 7.41	−17.10 ± 10.50	0.134
	ECV (%)	20.79 ± 3.65	27.06 ± 3.37^c,d^	31.11 ± 4.50^c,d^	<0.001
	ECV_age_ (%)^a^	23.95 ± 2.46	24.30 ± 2.22^d^	30.70 ± 1.23^d^	0.001
	Rest Perfusion (ml/min/g)	1.31 ± 0.20	1.05 ± 0.21	N/A	0.083
	Stress Perfusion (ml/min/g)	2.40 ± 0.34	1.28 ± 0.24^c^	N/A	0.004
	MPR	1.83 ± 0.3	1.35 ± 0.29^c^	N/A	0.007
	MPR_age_^b^	2.25 ± 0.35	1.24 ± 0.12^c^	N/A	0.048
	RestHR (1/min)	135.50 ± 15.27	114.22 ± 33.67	139.50 ± 28.36	0.128
	Stress HR (1/min)	170.00 ± 19.16	153.22 ± 33.13	N/A	0.198

LVEF, left ventricle ejection fraction; ECV, extracellular volume; EDV, end-diastolic volume; ESV, end-systolic volume; GLS, global longitudinal strain; MPR, myocardial perfusion reserve; HR, heart rate; N/A, not applicable.

^a^ Extracellular volume adjusted for age.

^b^ Myocardial perfusion reserve adjusted for age.

^c^ Comparison with healthy controls, *p* < 0.05.

^d^ Comparison between HFpEF and HFrEF, *p* < 0.05.

### Cardiac magnetic resonance findings

**Figure 4:**
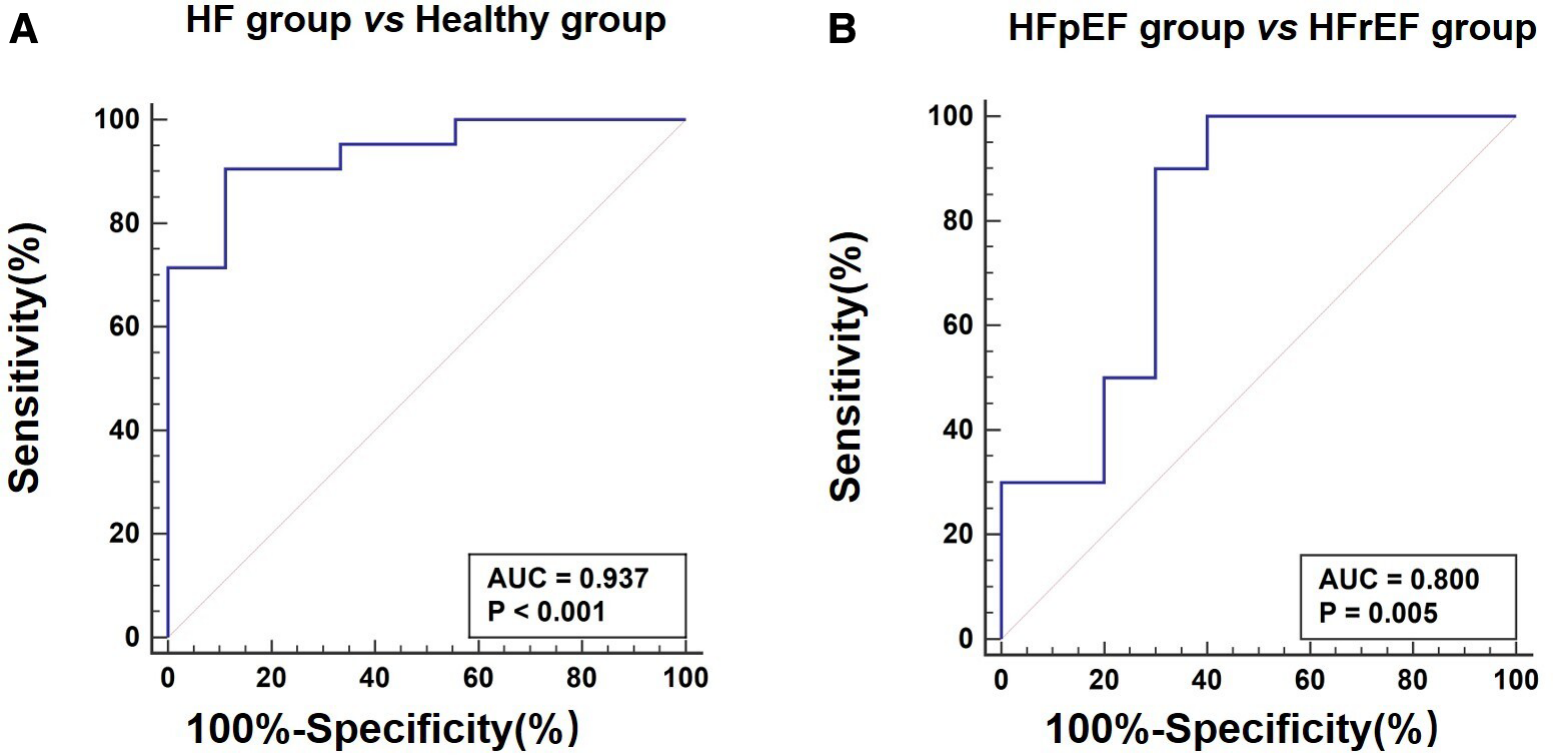
Receiver operating characteristic curves for extracellular volume fraction in heart failure. (**A**) Differentiation between heart failure group and healthy group. (**B**) Differentiation between HFpEF group and HFrEF group. HFpEF, Heart failure with preserved ejection fraction; HFrEF, Heart failure with reduced ejection fraction.

**Figure 5:**
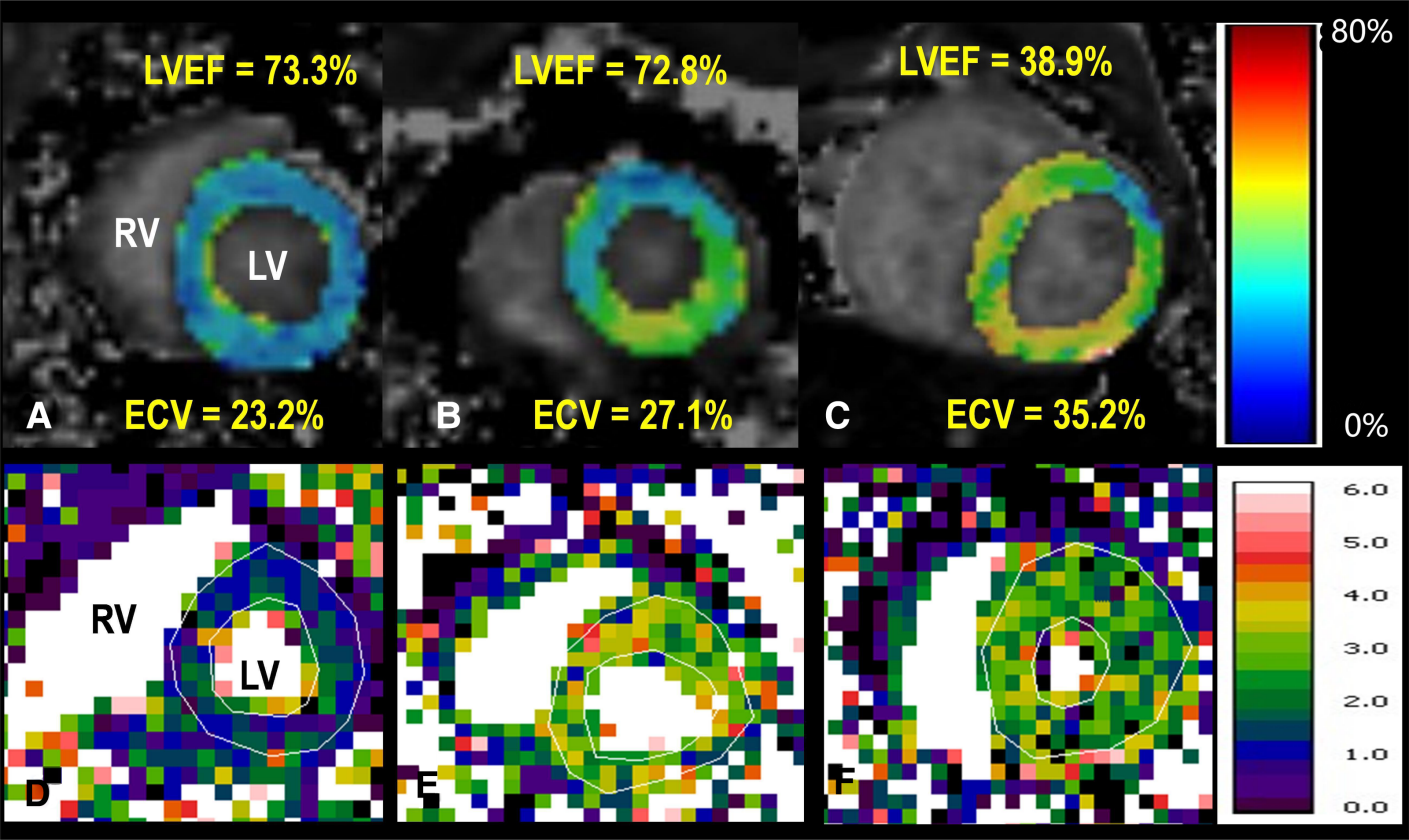
Top row: ECV maps of healthy (**A**), HFpEF (**B**), and HFrEF (**C**) Both global ECV and LV ejection fraction (LVEF) are listed. Bottom row: quantitative myocardial perfusion maps in a healthy monkey at rest (**D**) and during dobutamine (**E**), as well as a perfusion map of a monkey with HFpEF during dobutamine stress (**F**) The global perfusion values within enclosed myocardial regions are 0.93, 2.40, 1.44 ml/min/g, respectively. RV, right ventricle; LV, left ventricle. The color bar unit in bottom row is ml/min/g.

Table 3 lists LV function measured by echocardiography and CMR parameters (GLS, ECV, and perfusion) in the three groups of monkeys, clearly showing the differences in typical CMR biomarkers between the HF-like and healthy control groups. A decrease in cardiac function was associated with an increase in GLS (%) and ECV (%). There were significant differences in myocardial global ECV among the three groups (*p* < 0.001, *p*_adjusted-age _= 0.001), stress perfusion (*p* = 0.018, *p*_adjusted-age _= 0.123), and MPR (*p* = 0.011, *p*_adjusted-age _= 0.048). It was noteworthy that the age-adjusted ECV values were still statistically significant between the HFpEF and HFrEF groups, although they were no longer significantly different from the ECV of the healthy control group. The ECV correlated negatively with myocardial GLS (*r* = 0.67, *p* = 0.001), LVEF (*r *= 0.45, *p* = 0.03), E’/A’ (*r* = 0.60, *p* = 0.003), E’ (*r* = 0.57, *p* = 0.005), and E/A (*r* = 0.61, *p* = 0.002). After the adjustment for age, the myocardial global ECV was only found to be negatively correlated with LVEF (*r* = −0.69, *p* < 0.001). There was no LGE observed in any monkeys. The ECV exhibited apparent distinctions not only between the HF-like group and the healthy group (AUC = 0.937, *p* < 0.001), but also between the HFrEF group and the HFpEF group (AUC = 0.800, *p* = 0.005) (Figure 4). Figure 5 showed examples of ECV and MPR maps in three groups of monkeys.

## Discussion

In this study, the utilization of a high-fat diet to induce lipid metabolism disorder was employed to create a chronic HF-like NHP model. Our preliminary imaging findings showed HF-like features in the cardiac structure, function, and tissue characterization in these monkeys with both preserved and reduced EF. Diastolic dysfunction was clearly demonstrated by echocardiography, whereas the diffuse fibrosis (ECV) and perfusion determined by CMR exhibited similar abnormalities demonstrated in human patients with HF. The outcome of this research indicates that hyperlipidemia induced metabolic disorder could be a viable means for creating a HF-like NHP model. The use of the non-invasive CMR approach allows a reliable and sensitive assessment of myocardial tissue composition and perfusion. The current study thus may lay a solid foundation for building a HF-like NHP model for novel drug development and mechanism research in the future.

The most prevalent large animal HF models were dogs, pigs, sheep, cows, and NHPs (16–18). Several techniques were widely used to create heart failure according to the HF risk factors, including rapid cardiac pacing, coronary artery embolization, volume overload, pressure overload, hypertension, and metabolic pathway (diabetic/obesity) et al. (6). Although the metabolic pathway model cost more and took a longer time to develop, it was likely to better replicate the pathological progression of the HF disease. Obesity was an independent risk factor for the development of HF (19). Type 2 diabetes was an independent mortality predictor across all heart failure and the prevalence of diabetes in patients with heart failure ranges from approximately 25%–40% in humans (20, 21). The incidence rate of diabetes in the HF-like group of monkeys was apparently much higher than in human research, possibly due to the small sample size and the specific high-fat diet.

High-fat diet NHP models have been reported previously, in which increased oxygen consumption and/or cardiac contractile dysfunction was observed, along with increased expression of proinflammatory cytokines and altered phosphorylation of intracellular signaling proteins in myocardial tissue (22, 23). The cardiac structure, function, and myocardial fibrosis were investigated in studies with similar NHP models (15, 24). The current study, for the first time, provided quantitative information about the cardiac perfusion in this HF-like NHP model and the difference in the diffuse fibrosis content between the HFpEF and HFrEF groups.

The utilization of Doppler ultrasound has enabled the measurement of both *E*/*E*′ and *E*/*A* in various animal studies. The normal ranges for these parameters differ among animal species, with *E*/*E*′ typically ranging from 10 to 16 and *E*/*A* ranging from 1 to 2.5 (25–27). At present, there was no recognized heart failure threshold in the monkey model. We selected monkeys with diastolic dysfunction when at least two criteria were satisfied, which was developed based on our experience with monkey models. The reduced LVEF was defined as LVEF < 65% in cynomolgus monkeys, whereas healthy monkeys usually have LVEF ≥ 65%, which was apparently higher than the LVEF of healthy rhesus monkeys (∼57%) (15).

Multiple heart failure guidelines have given their approval for the use of CMR imaging in the diagnosis of heart failure (28, 29). The expansion of the extracellular matrix in the myocardium was a significant pathophysiological anomaly that was believed to be a primary contributor to the progression of HF (30). Previous studies have found that fibrosis index ECV correlated with diastolic dysfunction grades and adverse outcomes (31). In Rommel's study, 29 patients with HFpEF exhibited higher ECV compared to 12 controls without heart failure symptoms (32). In our study, ECV in monkeys with HFrEF was significantly higher than that in the healthy controls or in monkeys with HFpEF. The results matched well with ECV findings of diffuse fibrosis in patient studies, e.g., 27.8 ± 4.6% in HFpEF and 29.55 ± 1.45% in HFrEF (33, 34). Our average ECV in cynomolgus monkeys with HFpEF fell in the range of ECV reported in rhesus monkeys with HFpEF (26%–29%) (15).

It was noted that the ages were significantly different between the healthy and HF-like groups in this pilot study, even though all the subjects were adult monkeys (the rate of aging ratio of human to cynomolgus monkey years was approximately 1:4). There were conflicting reports about how age affects myocardial ECV (35, 36). This study would like to use the most healthy group, the young adult group, to determine the levels of elevated ECV in the HF-like groups. After adjusting for age in statistical analysis, the global ECV still showed a gradually increasing trend from healthy controls to HF-like monkeys. Furthermore, there was a statistically significant difference in global ECV values between the HFpEF and HFrEF groups. Age has been shown to play a significant role in the development of heart dysfunction (37). HFrEF prevalence strongly decreased whereas that of HFpEF strongly increased with aging (38). This was in line with our observation that monkeys in the HFpEF group with lower ECV values were older than those in the HFrEF group with higher ECV values. This also suggested that the difference in ECV values between two HF-like groups was largely due to the heart dysfunction itself. Future studies with more subjects were needed to define the age impacts.

Myocardial perfusion abnormality was highly prevalent in patients with HFpEF and was an independent factor of prognosis (39–41). Our perfusion measurements revealed similar perfusion features as in other studies in large animals and human patients, i.e., MPR was significantly lower in monkeys with HFpEF, compared to healthy controls (Table 3). For example, a study in a porcine model of HFpEF showed a reduced global myocardial perfusion reserve of 1.5 ± 0.4 with dobutamine stress (40, 42). In human studies, a reduced MPR was 1.74 ± 0.76 in patients with HFpEF, in comparison with 2.22 ± 0.76 in healthy controls with adenosine vasodilation (40). It is thus reasonable to assume that both diffuse fibrosis and impaired myocardial perfusion would be therapeutic targets in our HF-like NHP model.

There were several limitations in the present study. First, we have not conducted a comprehensive measurement of serum biomarkers, including BNP and NT-proBNP, as there was no suitable test kit for the monkey (43). This is an ongoing study with more innovative kits being tested. Second, the number of monkeys, especially monkeys with HF-like features, was limited. No reproducibility study was conducted since the imaging techniques used in this study were all well-established in the medical field. Third, an exercise intolerance test was not conducted in this pilot study. CMR functional scans were not performed to shorten the imaging time as much as possible without compromising the primary goal of this study, i.e., myocardial tissue characterization. Nevertheless, this preliminary study encouraged further expansion of this HF-like NHP model for more comprehensive phenotyping and imaging assessments. Finally, the histopathological assessment would provide a reference for validating the image findings. However, due to ethical and cost considerations, this study did not sacrifice any animals for tissue analysis. An alternative procedure could be done in the future using the biopsy approach.

## Conclusions

In this preliminary imaging study, a HF-like NHP model recapitulates major cardiac features of myocardial function, tissue characterization, and perfusion in human patients with preserved and reduced EF. While this HF-like NHP model needs more comprehensive assessments, the current image results will certainly serve as a stepping stone for further mechanistic research and the development of a drug testing platform for distinct HF pathophysiologies.

## Data Availability

The original contributions presented in the study are included in the article/Supplementary Material, further inquiries can be directed to the corresponding authors.
